# *Eremogone ali-gulii* (Caryophyllaceae), a new species from Turkey

**DOI:** 10.3897/phytokeys.61.7082

**Published:** 2016-02-25

**Authors:** Murat Koç, Ergin Hamzaoğlu

**Affiliations:** 1 Department of Biology, Faculty of Art & Sciences, Bozok University, 66100, Yozgat – Turkey; 2 Animal Production High School, Bozok University, 66100, Yozgat – Turkey; 3 Department of Elementary Education, Gazi Faculty of Education, Gazi University, 06500, Ankara - Turkey

**Keywords:** New species, *Eremogone*, Taxonomy, Turkey

## Abstract

*Eremogone ali-gulii* (Caryophyllaceae) is described as a new species of *Eremogone* in Turkey. The specimens were collected from Kop Mountain (Erzurum). The new species is endemic of the Irano-Turanian region and is related to *Eremogone scariosa* and *Eremogone armeniaca*. The differences on sterile shoots, habit, sepals and capsules between these species are discussed. Description, distribution, illustration and conservation status of the new species are given.

## Introduction

Caryophyllaceae is a very large family mainly found in the northern hemisphere and includes 88 genera and 3000 species (Rabeler and Hartman 2005). The family has often been divided into three subfamilies namely *Alsinoideae* Burnett, *Caryophylloideae* Arn., and *Paronychioideae* A.St. and five tribes (Bittrich 1993). However, recent molecular studies have illustrated that these groups are polyphyletic (Fior 2006). For instance, Harbaugh et al. (2010) evaluated the *Mat*K, *trn*L-F and *rps*16 sequences of 146 species of Caryophyllaceae and recognized 11 tribes, including the newly described *Eremogoneae* Rabeler & W.L.Wagner (Harbaugh et al. 2010).

*Eremogone* was described by Fenzl in 1833, then it was later described as a subgenus of *Arenaria* by Fenzl in 1842. Molecular phylogenetic studies carried out in recent years suggest that species previously placed in Arenaria subgen. Eremogone (approximately 70 species), Arenaria subgen. Eremogoneastrum Williams (22 species) and Minuartia subgen. Spergella (Fenzl) McNeill (3 species) comprise the genus *Eremogone* (Fenzl 1833, McNeill 1967). *Eremogone* is a natural group consisting of 95 species with leaves narrowly linear to filiform and capsules splitting apically into six teeth or three bifid valves (Dillenberger and Kadereit 2014, Sadeghian et al. 2015). The Turkish name for *Eremogone* is “İğnekumotu”. There are 18 taxa of *Eremogone* in Turkey, 11 of which are endemic (McNeill 1967, Dinç 2012). In the flora of Turkey (McNeill 1967), *Eremogone* is treated as a subgenus of *Arenaria* (Fenzl 1833, McNeill 1967). This work describes *Eremogone ali-gulii*, a new species of *Eremogone* found in Turkey.

## Material and methods

Authors collected *Eremogone* specimens from Kop mountain (Erzurum) during a project (KBAG-113Z260-TUBITAK) to revise Turkey *Minuartia* taxa (Caryophyllaceae). These specimens were compared with related species or photographs in the herbaria of E, ANK, GAZI, Bozok University Herb., and with records in the literature (McNeill 1963, Rechinger 1964, Zohary 1966, McNeill 1967, Halliday 1976, Rechinger 1988, Shishkin 1995, Bojňanský and Fargašová 2007, Rabeler and Wagner 2015). The studies showed that these specimens are representatives of a species new to science.

This study is based on literature and field observations of living plants. The materials were examined using an Olympus SZ61 microscope. Mature seeds were collected from capsules of the holotype. Measurement of vegetative characters was made with a ruler accurate to 0,5 mm and floral characters were measured with an ocular micrometre.

## Taxonomic treatment

### Eremogone ali-gulii

Taxon classificationPlantaeCaryophyllalesCaryophyllaceae

Koç & Hamzaoğlu sp. nov.

urn:lsid:ipni.org:names:77153392-1

Fig. 1

#### Diagnosis.

*Eremogone ali-gulii* is similar to *Eremogone scariosa* (Boiss.) Holub and *Eremogone armeniaca* (﻿Boiss.) Holub, but differs in having fasciculate sterile shoots, a tufted habit and shorter sepal and capsules.

**Figure 1.**
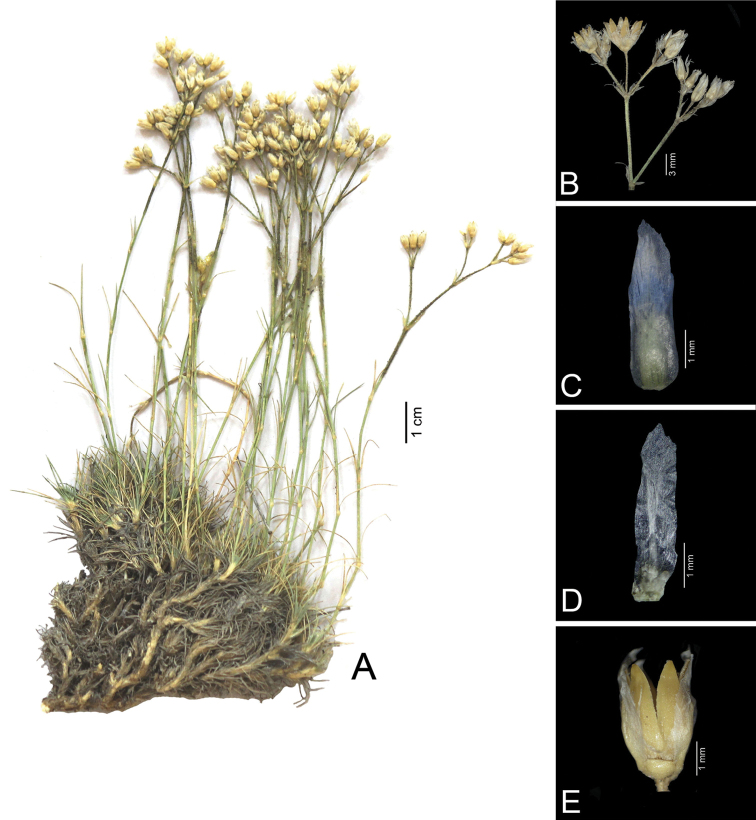
*Eremogone ali-gulii* (Koç 1723). **A** Habit **B** Inflorescence **C** Sepal **D** Petal **E** Capsule.

#### Type.

TURKEY, Erzurum province, between Bayburt and Aşkale, Kop mountain, 40°00'N-040°32'E, 2150 m, serpentine stony slopes, 24 June 2014, Koç 1723 & Hamzaoğlu (holotype ANK, isotype Bozok Univ. Herb., ANK, GAZI).

#### Description.

Tufted, perennial herb. Stems erect or ascending, 10–18 cm long and 0.7–0.9 mm in diameter, glabrous below, glandular-puberulent above. Rosette leaves setaceous, 1.5–2.5 cm, fasciculate; stem leaves linear-setaceous, 0.6–15 × 0.5–0.8 mm, smooth, glabrous, 3–5 pairs, apex acute to acuminate, leaf sheath membranous, 0.1–0.2 mm, glabrous. Bracts oblong-lanceolate, 2.5–5 × 0.8–1.2 mm, glandular hairy, obscurely 3-nerved, apex acute to acuminate. Inflorescences usually terminal, 3–10-flowered, panicles, the peduncles and pedicels sparsely to densely glandular-puberulent; bracts oblong-lanceolate, 4–6 × 0.5–0.8 mm, apex setaceous-acuminate, margins scarious never extending right to the tip; pedicels 1–4 mm long. Flower sepals oblong-lanceolate, 2.8–4.5 × 1.2–1.7 mm, glabrous, obscurely veined, membranous at the base, scarious above, the apex obtuse to acute; petals white, linear-oblong, 3–5 × 0.8–1.2 mm, slightly longer than sepals, the apex obtuse to acute; stamens 10, the filaments 2–3.5 mm long; styles 3, more or less erect, 1.5–2 mm long; staminal glands deeply bifurcate, appearing as 10, distinct, alternating with the stamens. Capsules 2.5–4 × 0.8–1.2 mm, ovoid to elongate-ovoid, glabrous, opening by 6 recurved teeth, containing only a few seeds. Seeds 1.9–2.4 mm long, oblong, tuberculate on the rim and the sides with low, elongate tubercles, black to dark brown. Flowering in June and July.

#### Ecology.

*Eremogone ali-gulii* grows on serpentine, which is the most widespread of the non-calcareous soils in between the cities of Erzurum and Erzincan in Turkey. It occurs in stony slopes habitat with *Achillea biebersteinii* Afan, *Pimpinella rhodantha* Boiss., *Inula heterolepis* Boiss., *Arenaria pseudoacantholimon* Bornm., *Arenaria serpyllifolia* L., Dianthus crinitus Sm. var. crossopetalus Boiss., Helichrysum plicatum DC. subsp. polyphyllum (Ledeb.) Davis & Kupicha.

#### Conservation status.

The species is currently known from two populations: in the location of Kop mountain in Erzurum and another location between Erzurum-Erzincan, around Karasu village. Both populations are vulnerable to anthropogenic impact. Informal grazing and land-use changes could have a detrimental impact in the future. On the basis of IUCN red list categories and criteria (IUCN 2012), *Eremogone ali-gulii* covers an area (AOO) of about 300 km^2^. The new species is here assessed as Endangered [EN, B2ab(iii)].

#### Etymology.

The species is named in honour of the eminent Turkish hydrobiologist Prof. Dr. Ali Gül (Gazi Faculty of Education, Gazi University, Ankara).

## Results

The specimens introduced here as the new species in this study were collected from Erzurum province, Kop mountain. At first glance, these specimens resemble *Eremogone armeniaca* and *Eremogone scariosa*. Yet, comprehensive studies that were subsequently carried out revealed that they belonged to a new species.

### Distinction from other taxa

*Eremogone ali-gulii* is similar to a group of five Irano-Turanian *Eremogone* species previously placed in Arenaria sect. *Scariosae* by McNeil (1967): 2 from Northern Iran - *Eremogone polycnemifolia* (Boiss.) Holub and *Eremogone zargariana* (Parsa) Holub; and 3 species from eastern Turkey - *Eremogone armeniaca*, *Eremogone scariosa* and *Eremogone pseudoacantholimon* (Bornm.) Holub. All of them share spiny or setaceous leaves, coriaceous or scarious sepals, and petals of similar size. *Eremogone ali-gulii* is more similar to *Eremogone armeniaca* and *Eremogone scariosa* due to the scarious margins of the sepals never extending right to the tip and to the deeply bifurcate staminal glands, appearing as 10, distinct, alternating with the stamens (McNeill 1962). However, *Eremogone ali-gulii* differs markedly from both species due to its fasciculate sterile shoots, tufted habit, sepals 2.8-4.5 mm long and capsules 3-4 mm long. The diagnostic features of these three species are listed in Table 1.

**Table 1.** Diagnostic characters of *Eremogone ali-gulii* compared with other similar species.

Characters	*Eremogone ali-gulii*	*Eremogone armeniaca*	*Eremogone scariosa*
Habit	tufted	suffruticose	suffruticose
Stem	10–18 cm long	20–30 cm long	10–20 cm long
Rosette leaves	fasciculate	imbricate	imbricate
Inflorescence	panicle	terminal cluster enclosed by glumaceous bracts	panicle
Pedicels	2–4 mm long	1–2 mm long	2–6 mm long
Sepals	2.8–4.5 mm long, membranous at the base, scarious at the apex	(4.5)6–9 mm long, membranous at the extreme apex	6–8 mm long, membranous at the base, scarious at the apex
Petal shape	linear-oblong	oblanceolate	linear-oblong
Capsule	3–4 mm long	4–7 mm long	5–7 mm long

### Key to closely related *Eremogone* species

**Table:** 

1	Sepals with coriaceous herbaceous median strip, very gradually narrowing to the tip; staminal glands 5, indistinct, at the base of the outer whorl of stamens (Series *Polycnemifoliae*)	
–	Sepals with scarious margins never extending right to the tip; staminal glands deeply bifurcate, appearing as 10, distinct, alternating with the stamens (Series *Scariosae*)	
2	Sepals 6–8 mm long; bracts 7–8 mm long	***Eremogone zargariana***
–	Sepals 3.5–5.5 mm long; bracts 3–5 mm long	
3	Stem leaves 1–2 pairs; rosette leaves 1–2 cm, stiff, aristate; sepals ovate-lanceolate; petals as long as sepals	***Eremogone pseudacantholimon***
–	Stem leaves 3–9 pairs; rosette leaves 10–15 cm, not stiff, acute to acuminate; sepals oblong-lanceolate; petals slightly longer than sepals	***Eremogone polycnemifolia***
4	Habit tufted; sterile shoots fasciculate, sepals 2.8–4.5 mm long; capsules 3–4 mm long	***Eremogone ali-gulii***
–	Habit suffruticose; sterile shoots imbricate, sepals (4.5)6–9 mm long; capsules 4–7 mm long	
5	Inflorescence a terminal cluster enclosed by glumaceous bracts; sepals membranous at the extreme apex; petals slightly longer than sepals	***Eremogone armeniaca***
–	Inflorescence paniculate; sepals membranous at the base, scarious above; petals slightly shorter than sepals	***Eremogone scariosa***

### Specimens examined

***Eremogone ali-gulii*** (Paratype), TURKEY - B8 Erzurum: between Erzurum-Erzincan, Karasu village around, 1600 m, 15.06.2007, E.Hamzaoğlu 4662 (Bozok Univ. Herb.!). – ***Eremogone scariosa*** (Boiss.) Holub, TURKEY - B5 Bayburt: Between Bayburt and İspir, Karşıgeçit village, 1460 m, 12.07.2009, Koç 589, Ü.Budak and E.Hamzaoğlu (Bozok Univ. Herb.!); Bayburt: SE of Bayburt, 1500-1700 m, 13.06.2002, E.Hamzaoğlu 2960 (Bozok Univ. Herb.!); Bayburt: Between Bayburt and Pazaryolu, 23.06.2002, E.Hamzaoğlu 3009 (Bozok Univ. Herb.!); Gümüşhane: Yukarıalıçlı village, 1500 m, 14.07.2007, E.Hamzaoğlu 4819 and A.Aksoy (Bozok Univ. Herb.!); A7 Gümüşhane: Kovans, ca. 1800 m, 02.08.1957, P.H.Davis and I.C.Hedge (ANK-31937); A8 Bayburt: Darıca village, Çoruh valley, 1720-1850 m, 26.08.1991, T.Ekim, M.Koyuncu, H.Karaca and A.Güner 9707 (GAZI!); A7 Gümüşhane: Köse-Gümüşhane bei Kirikli, 1300 m, 12.07.1984, M.Nydegger (E, E00074746-photo!). – ***Eremogone armeniaca*** (Boiss.) Holub, TURKEY - A6 Sivas: Zara-Suşehri, 20 km from Suşehri, 1300 m, 23.07.1960, A.Stainton and D.M.Henderson (E, E00567097-photo!); A7 Giresun: N of Şebinkarahisar, 40°20'N-038°26'E, 24.07.2010, Hamzaoğlu 5896 and Koç (Bozok Univ. Herb.!); A7 Gümüşhane: Sorda, nr. Teke, P.E.E.Sintenis 1894 (E, E00567094-photo!); A8 Erzurum: Tortum around, 1500 m, 17.07.1990, Z.Aytaç 3149, T.Ekim and H.Duman (GAZI!); A8 Erzurum: Tercan-Ilıca, 1900 m, 10.07.1957, P.H.Davis 30843 and I.C.Hedge (E, E00567095-photo!); A8: Bayburt: Maden, between Masat-Yanıkköprü, 1910 m, 17.07.2007 Hamzaoğlu 4893 and Aksoy (Bozok Univ. Herb.!); A8 Erzurum: W of Kandilli, 1520 m, 17.08.1966, J.C.Archibald (E, E00567098-photo!); B7 Erzincan: Erzincan to Kelkit, 1650 m, P.H.Davis 31885 and I.C.Hedge (E, E00567096-photo!); B7 Erzincan: Between Tercan-Aşkale, Yakacık village, 1570 m, 02.07.2013 Hamzaoğlu 6786 and Koç (Bozok Univ. Herb.!); B8 Erzurum: Between Erzurum-İspir, 40°09'N-041°01'E, 2010 m, 02.07.2013, Hamzaoğlu 6788 and Koç (Bozok Univ. Herb.!). – ***Eremogone pseudacantholimon*** Bornm., TURKEY - B7 Erzincan: between Erzincan-Refahiye, 39°55'N-039°08'E, 24.08.2012, Hamzaoğlu 6623 and Koç (Bozok Univ. Herb.!); B7 Erzincan: between Erzincan-Kelkit, 39°52'N-039°23'E, 2095 m, 13.07.2010, Koç 1277 and Hamzaoğlu (Bozok Univ. Herb.!); B7 Tunceli: between Tunceli-Pülümür, 39°31'N-039°52'E, 1850 m, 09.06.2011, Koç 1330 and Hamzaoğlu (Bozok Univ. Herb.!); B8 Erzurum: between Çat-Bingöl, Kirişli pass, 2320 m, 16.07.2007, Hamzaoğlu 4863 and Aksoy (Bozok Univ. Herb.!). – ***Eremogone polycnemifolia*** Boiss., IRAN – Shahkuh, Mazenderan, 10000 ft, 17.07.1940, W.Koelz (E, E00194277-photo!); Arak: Streamside and stagnant pools, 25.07.1963, N.Jardine (E, E00567090-photo!); Tehran: Elburz mts, W. of Firuzkuh, 7000 ft, 23.06.1960, P.Furse and P.Synge (E, E00567089-photo!); Mazandaran: Lar valley, by Lar river, 2500 m, 03.07.1974, P.Wendelbo and M.Assadi (E, E00567089-photo!). – ***Eremogone zargariana*** Boiss., IRAN – Montes Elburz: In saxosis summi montis Kuh Dashteh c. 30 km Tehran, 2400-2500 m, 28.06.1977, K.H.Rechinger (E, E00567093-photo!); Tehran: East of Rudehan, semi-desert country, 1520 m, 03.07.1966 J.C.Archibald (E, E00567092-photo!).

## Supplementary Material

XML Treatment for Eremogone ali-gulii

## References

[B1] Bittrich V (1993) Caryophyllaceae. In: Kubitzki K Rohwer J Bittrich V (Eds) The Families and Genera of Vascular Plants, Magnoliid, Hamamelid, and Caryophyllid Families, Vol. 2 Springer, Berlin, 206–236. doi: 10.1007/978-3-662-02899-5_21

[B2] Bojňanský V Fargašová A (2007) Atlas of seeds and fruits of Central and East-European Flora: The Carpathian Mountains Region. Springer, Netherlands, 47–91.

[B3] Dillenberger MS Kadereit JW (2014) Maximum polyphyly: Multiple origins and delimitation with plesiomorphic characters require a new circumscription of *Minuartia* (Caryophyllaceae). Taxon 63: 64–88. doi: 10.12705/631.5

[B4] Dinç M (2012) *Eremogone* Fenzl. In: Güner A Aslan S Ekim T Vural M Babaç MT (Eds) Türkiye Bitkileri Listesi (Damarlı Bitkiler). Nezahat Gökyiğit Botanik Bahçesi ve Flora Araştırmaları Derneği Yayını, İstanbul, 337–339.

[B5] Fenzl E (1833) Versuch einer Darstellung der Geographischen Verbreitungs- und Vertheilungs-Verhältnisse der natürlichen Familie der Alsineen 13, and unnumbered plate.

[B6] Fior S Karis PO Casazza G Minuto L Sala F (2006) Molecular phylogeny of the Caryophyllaceae (Caryophyllales) inferred from chloroplast matK and nuclear rDNA ITS sequences. American Journal of Botany 93: 399–411. doi: 10.3732/ajb.93.3.399 21646200 10.3732/ajb.93.3.399

[B7] Harbaugh DT Nepokroeff M Rabeler RK McNeill J Zimmer EA Wagner WL (2010) A new lineage-based tribal classification of the family Caryophyllaceae. International Journal of Plant Science 171: 185–198. doi: 10.1086/648993

[B8] IUCN Red List Categories and Criteria: Version 3.1 Second edition IUCN, Gland, Switzerland and Cambridge, UK, iv + 32 pp.

[B9] McNeill J (1962) Taxonomic studies in the Alsinoideae. A revision of the species in the Orient. Notes from the Royal Botanical Garden Edinburgh 24(1): 102–140.

[B10] McNeill J (1967) *Arenaria* L. In: Davis PH (Ed.) Flora of Turkey and the East Aegean Islands, Vol. 2 Edinburgh University Press, Edinburgh, 17–38.

[B11] Rabeler RK Hartman RL (2005) Caryophyllaceae in Flora of North America North of Mexico, vol. 5 Oxford University Press, New York, 3–8.

[B12] Rabeler RK Wagner WL (2015) *Eremogone* (Caryophyllaceae): new combinations for Old World species. Phytokeys 50: 35–42. doi: 10.3897/phytokeys.50.4736 26140019 10.3897/phytokeys.50.4736 PMC4489083

[B13] Rechinger KH (1964) *Arenaria* L. In: Rechinger KH (Ed.) Flora of Lowland Iraq. Weinheim, Austria, 230–231.

[B14] Rechinger KH (1988) *Arenaria* L. In: Rechinger KH (Ed.) Flora Iranica (Caryophyllaceae II). Akademische Druck-u Verlagsanstalt, Austria, 6–28.

[B15] Sadeghian S Zarre S Rabeler RK Heubl G (2015) Molecular phylogeny of *Arenaria* (Caryophyllaceae: tribe Arenarieae) and its allies inferred from nuclear DNA ITS and plastid DNA rps16 sequences. Botanical Journal of the Linnaean Society 178: 648–669. doi: 10.1111/boj.12293

[B16] Shishkin BK (1995) *Arenaria* L. In: Shishkin BK (Ed.) Flora of the U.S.R.R., Vol. 6 Bishen Singh Mahendra Pal Singh and Koeltz Scientific Books (English version), Moskva-Leningrad, 398–414.

